# The dead still teach the living: Body donor dissection in the digital age of anatomy education

**DOI:** 10.1002/ase.70315

**Published:** 2026-08-10

**Authors:** Uğur Baran Kasırga

**Affiliations:** ^1^ Department of Anatomy, Faculty of Medicine Bahçeşehir University Istanbul Türkiye

**Keywords:** anatomical variation, anatomy education, artificial intelligence, body donation, clinical reasoning, digital anatomy, donor respect, donor‐based dissection, medical education, professional identity, simulation, three‐dimensional learning

## Abstract

Donor‐based dissection has become increasingly contested in modern medical curricula as digital anatomy platforms, simulation technologies, virtual dissection tables, three‐dimensional reconstructions, and artificial intelligence‐supported learning have expanded. Reduced curricular time and logistical constraints have encouraged some institutions to reduce or replace dissection‐based anatomy teaching. This shift raises a central educational question: What does dissection contribute to anatomy learning that cannot be reproduced by other methods? This article examines donor‐based dissection from an anatomy‐education perspective. It argues that its value should not rest on tradition, nor be judged solely by examination performance. Its educational significance lies in the multiple dimensions of learning it uniquely supports: three‐dimensional spatial reasoning, recognition of anatomical variation, tactile and manual understanding of tissue planes, regional and topographical orientation, integration of structure with clinical relevance, and the development of professional identity and donor respect. Digital anatomy and artificial intelligence can enrich anatomy teaching by improving visualization, accessibility, and repetition. However, these tools present anatomy in a standardized, cleaned, and immediately visible form. Donor‐based dissection exposes students to the body as layered, variable, and sometimes ambiguous—an encounter that cannot be fully replicated digitally. A six‐phase hybrid model grounded in experiential learning theory is proposed: digital pre‐laboratory orientation, donor‐based or prosection‐based exploration, imaging correlation, clinical case reasoning, guided professional reflection, and post‐laboratory digital consolidation. In this framework, digital tools prepare and reinforce learning while dissection remains the central embodied experience. Donor‐based dissection therefore remains indispensable as a carefully structured component of an integrated anatomy curriculum.

## INTRODUCTION: REFRAMING THE QUESTION FOR ANATOMY EDUCATION

For centuries, dissection of the human body has represented one of the defining experiences of medical education.^1^, ^2^, ^3^ The dissection room has served not only as a place where students learn the arrangement of muscles, vessels, nerves, organs, and fascial planes but also as a formative environment in which they encounter the human body in its material reality. Yet the place of dissection in the medical curriculum has repeatedly been questioned. The reasons are understandable. Modern curricula are increasingly crowded, integrated, system‐based, and competency‐oriented.^3^, ^4^, ^5^, ^6^ Anatomy often competes for curricular time with molecular medicine, imaging, communication skills, evidence‐based practice, clinical skills, ethics, and early patient contact. At the same time, donor‐based anatomy teaching requires donor programs, laboratory facilities, preservation systems, technical staff, trained educators, institutional governance, and substantial financial investment.

The debate has intensified with the rapid development of alternative educational technologies. Today, students can study anatomy through high‐quality atlases, sectional imaging, interactive three‐dimensional platforms, virtual dissection tables, augmented and virtual reality, ultrasound, simulation laboratories, 3D‐printed models, online videos, adaptive question banks, and artificial intelligence‐supported tutoring systems.^7^, ^8^ These tools have clear educational value. They improve access, allow repetition, standardize visualization, support self‐paced learning, and enable students to revisit difficult regions outside the laboratory. They also allow anatomy to be linked more easily with imaging, clinical cases, and assessment.

However, the availability of alternatives does not automatically establish educational equivalence. The fact that anatomical information can be displayed digitally does not mean that all forms of anatomical understanding can be achieved digitally.^2^ The essential question is therefore not whether anatomy can be taught without dissection in the narrow sense. Many anatomical facts can certainly be learned without entering a dissection room. The more important question is which dimensions of anatomical learning are uniquely developed through donor‐based dissection, and how these dimensions should be incorporated into contemporary medical curricula.

This article reframes the debate from an anatomy‐education perspective. The central issue is not whether donor‐based dissection should be preserved because it is traditional, but whether it provides forms of anatomical learning that remain pedagogically necessary. Its value lies in the way it brings together spatial reasoning, tactile experience, anatomical variation, regional orientation, clinical localization, teamwork, ethical awareness, and professional identity.^2^, ^9^ Digital anatomy, simulation, and artificial intelligence should not be treated as enemies of dissection. Rather, they should be used to prepare students for learning on donated bodies, reinforce it, and translate it into clinical reasoning. The future of anatomy education should therefore not be solely donor‐based or solely digital; it should be pedagogically integrated, clinically meaningful, and anatomically realistic (Figure 1).

**FIGURE 1:**
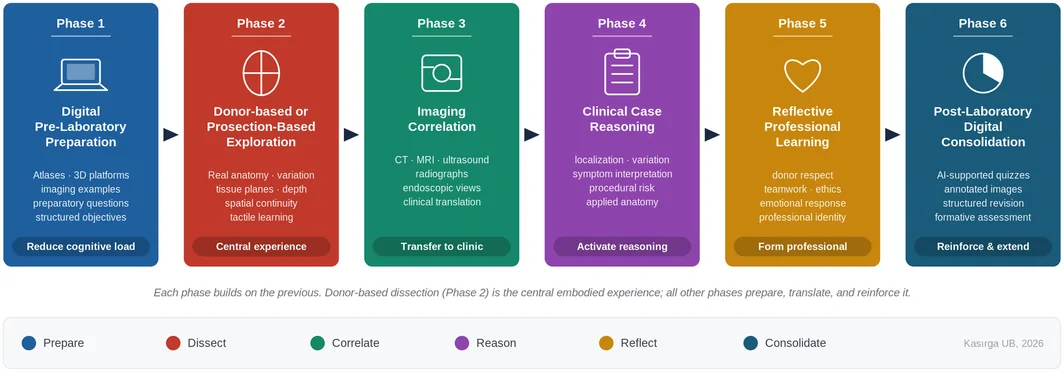
Hybrid model for contemporary anatomy education. Digital pre‐laboratory preparation → Donor‐based or prosection‐based exploration → Imaging correlation → Clinical case reasoning → Reflective professional learning → Post‐laboratory digital consolidation. Donor‐based dissection (Phase 2) is the central embodied experience; all other phases prepare, translate, and reinforce it.

## ANATOMY EDUCATION UNDER CONTEMPORARY PRESSURE

The questioning of donor‐based dissection cannot be understood without considering the broader transformation of medical education. Traditional discipline‐based curricula often allocated extensive time to gross anatomy in the early years of medical school. Students learned anatomy as a foundational science, commonly through lectures, atlases, dissection, and practical examinations. In many contemporary curricula, this model has been replaced or modified by integrated system‐based teaching, problem‐based learning, case‐based learning, early clinical exposure, and competency‐based frameworks.^3^, ^4^ These reforms have many strengths, but they have also compressed the time available for systematic anatomical study.^5^, ^6^

This compression has important consequences. Anatomy is not a subject that can be fully mastered through brief exposure. It requires repeated spatial orientation, integration across regions, and progressive reinforcement through clinical application. When teaching time is reduced, there is a temptation to replace complex laboratory experiences with faster and more standardized digital content. Such replacement may appear efficient, especially when institutions face increasing student numbers, decreased teaching time, limited access to body donors, high maintenance costs, and growing administrative expectations.^4^, ^5^, ^6^, ^7^

Donor‐based anatomy teaching also presents broader systemic challenges that extend beyond individual institutions. Globally, access to body donation programs is highly unequal.^4^, ^6^ In many low‐ and middle‐income settings, the infrastructure required for a functioning donor program—including legal frameworks, preservation facilities, trained staff, and institutional governance—may not be available or sustainable. Even in well‐resourced institutions, donor shortages are a recognized constraint, and environmental concerns related to chemical preservation have prompted interest in alternative approaches.^10^, ^11^ Increasing student cohort sizes place further pressure on the ratio of students to available donated material, raising questions about pedagogical equity and learning quality. These are structural challenges that must be honestly acknowledged in any discussion of donor‐based anatomy education (Figure 2).

**FIGURE 2:**
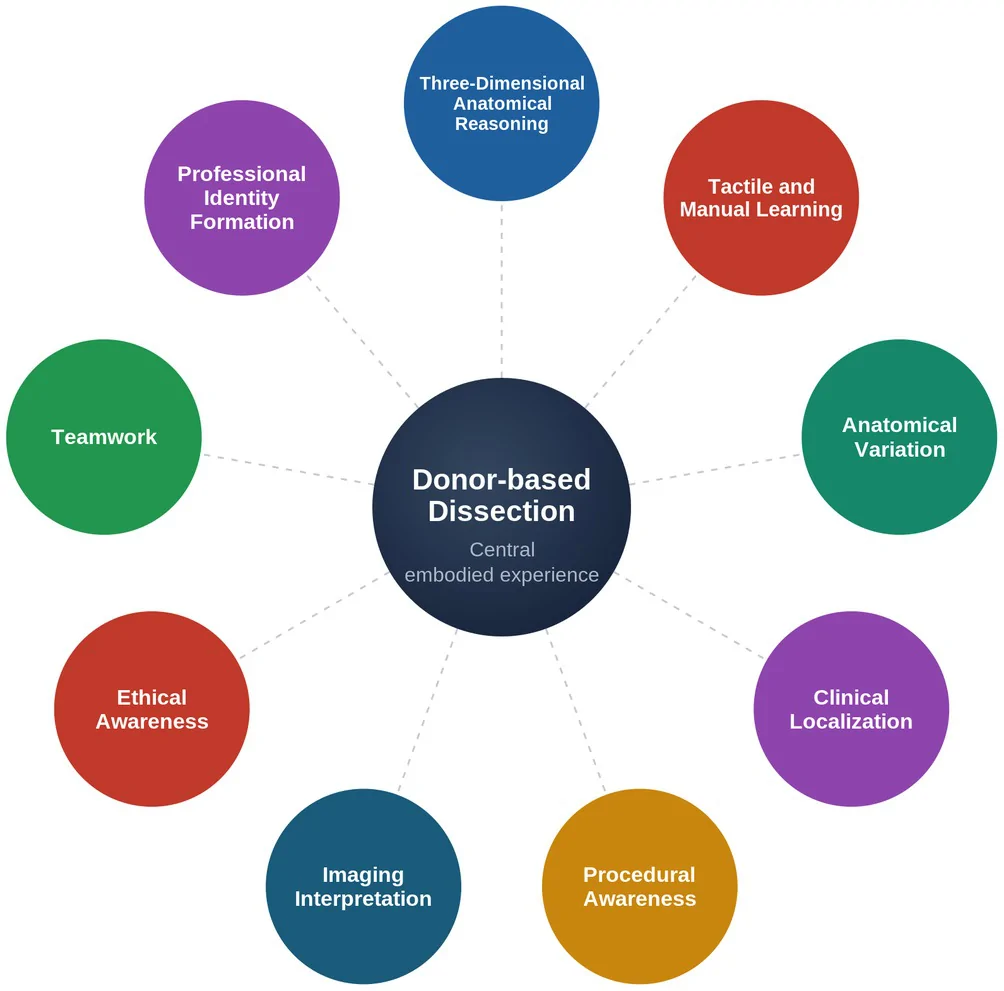
Dimensions of learning supported by donor‐based dissection. Donor‐based dissection contributes to multiple domains of medical education: Three‐dimensional anatomical reasoning, tactile and manual learning, recognition of anatomical variation, clinical localization, procedural awareness, imaging interpretation, ethical awareness, teamwork, and professional identity formation.

Donor‐based teaching also presents logistical and ethical responsibilities. A functioning dissection program requires legally and ethically governed body donation, appropriate facilities, health and safety measures, preservation expertise, trained technical personnel, and qualified anatomy educators.^10^ It also requires careful student preparation. For some learners, the first encounter with a donated body may be emotionally challenging.^12^, ^13^ These are real constraints, and they should not be dismissed.

At the same time, the growth of digital technologies has changed what students and faculty expect from anatomy education. Digital resources can show anatomical structures in clean, labeled, rotatable, and repeatable forms. They can display vessels, nerves, organs, bones, and muscles in isolation or in combination. They can integrate CT, MRI, ultrasound, and endoscopic perspectives. They can help students rehearse anatomy before the laboratory and revise it afterward. Artificial intelligence tools can generate explanations, quizzes, summaries, and personalized learning pathways.^14^ In this environment, donor‐based dissection must justify its role not by tradition alone, but by educational necessity.

The central issue is therefore not whether digital tools are useful. They clearly are. The issue is whether they can fully replace the educational experiences created by the encounter with the real human body.^2^, ^8^ To answer this, dissection must be analyzed not merely as a method for visualizing structures, but as a complex pedagogical environment.

## WHAT MAKES DONOR‐BASED DISSECTION EDUCATIONALLY UNIQUE?

Donor‐based dissection is often described as a way of seeing anatomy. This description is incomplete. Dissection is not only a visual method; it is a process of discovery.^2^ In a textbook, atlas, or digital model, anatomy is usually preselected, cleaned, colored, labeled, and idealized. The student is shown the structure after the intellectual and manual work of exposing it has already been completed. In dissection, by contrast, the student must encounter anatomy as it exists in the body: layered, variable, hidden, continuous, and sometimes ambiguous.

A donated body does not present itself as a diagram. It does not immediately reveal the nerve, vessel, fascia, organ, or plane of interest. Students must move from the surface to the depth. They must separate skin, fascia, fat, muscle, vessels, nerves, and organs while preserving meaningful relationships. They must learn that structures are not isolated labels, but parts of regions. They must recognize that visibility is produced through anatomical approach. This process teaches anatomy in a way that is fundamentally different from passive observation.^2^, ^9^

The educational value of dissection lies partly in this productive struggle. Students ask where a structure should be, what tissue plane should be followed, what must be preserved, what can be reflected, and how one structure relates to another. This transforms anatomy from a list of terms into a spatial and procedural logic.^10^ The student does not merely learn that the femoral nerve lies lateral to the femoral artery or that the recurrent laryngeal nerve ascends in the tracheoesophageal groove. The student learns how such relationships are embedded in tissues, fascial planes, and regional anatomy.

Dissection also introduces uncertainty. Real anatomy is not always immediately clear. Structures may be obscured by fat, altered by age, affected by pathology, distorted by preservation, or different from textbook expectations. This uncertainty is pedagogically valuable because clinical practice is also uncertain.^8^ A surgeon, radiologist, anesthesiologist, emergency physician, or general clinician rarely encounters anatomy in an idealized form. Dissection therefore prepares students for the intellectual discipline of anatomical interpretation. The capacity to tolerate and work through such uncertainty is itself an educational outcome of considerable value, one that digital models cannot easily replicate.^15^

## DISSECTION AND THREE‐DIMENSIONAL ANATOMICAL REASONING

One of the greatest difficulties in anatomy learning is the transition from two‐dimensional representation to three‐dimensional understanding.^16^ Many students can memorize structures from atlas plates but struggle to understand their spatial relationships in the body. They may know the names of muscles in a compartment, but not their depth, orientation, fascial boundaries, neurovascular supply, or relationship to adjacent regions. They may identify an organ in isolation but fail to appreciate its syntopy, projection, mobility, and clinical accessibility.

Donor‐based dissection directly addresses this problem. It requires students to think in three dimensions. They must understand what is superficial and what is deep, what is medial and lateral, what lies anterior or posterior, what crosses what, and how one region continues into another. They learn that the body is organized not as separate textbook chapters but as continuous anatomical territory.^2^ The axilla opens toward the neck and upper limb; the posterior abdominal wall is related to the kidneys, ureters, vessels, lumbar plexus, diaphragm, and psoas major; the pelvis and perineum are connected through fascial planes, neurovascular pathways, and visceral supports.

This spatial reasoning is especially important for topographical anatomy. Clinical practice depends on knowing not only the names of structures but also their positions, relations, projections, and access routes. A clinician must understand where a lesion is likely to be, which structures may be affected, how a pathology may spread, where a needle or incision may pass, and why a symptom appears in a particular distribution. Dissection provides a physical environment for constructing this internal anatomical map.^10^, ^17^

Digital models and virtual reality platforms can also support spatial learning, and research has demonstrated measurable improvements in students' spatial ability following use of interactive three‐dimensional anatomy software.^16^ Virtual reality and augmented reality tools offer the advantage of repeatable, standardized spatial exploration and may be particularly effective for students with initially lower spatial ability.^16^ These are genuine educational contributions that deserve recognition. However, the donated body adds a dimension of complexity that current digital tools cannot fully replicate: real depth, tissue continuity, tactile resistance, and anatomical ambiguity. In dissection, the student experiences the fact that spatial knowledge is not abstract. It is embodied in actual thickness, resistance, obscurity, and orientation. The two approaches are therefore complementary rather than competing; the aim should be to harness both within an integrated educational framework rather than to frame them as alternatives.

## DISSECTION AND ANATOMICAL VARIATION

Another essential contribution of donor‐based dissection is the direct encounter with anatomical variation. Atlases and digital models often represent a standardized body. This is useful for initial learning, but it can unintentionally create the impression that anatomy is fixed, symmetrical, and predictable.^18^, ^19^ In reality, human anatomy is variable. Arteries branch differently, veins may follow unexpected routes, nerves may communicate or divide atypically, muscles may have accessory slips, organs may vary in position, and fascial planes may be influenced by age, body habitus, disease, or previous surgery.

This variation is not a peripheral curiosity. It has direct clinical significance.^18^, ^19^, ^20^ Surgical approaches, vascular interventions, regional anesthesia, emergency procedures, imaging interpretation, and physical examination all require awareness that the patient may not conform exactly to the diagram. A clinician who has only learned idealized anatomy may be less prepared for unexpected findings. Dissection teaches students that variation is normal, not exceptional—a principle now supported by formal curriculum frameworks that treat anatomical variation as a core educational theme rather than an addendum.^19^

The educational impact of variation is particularly valuable because it is memorable. When students discover an unusual arterial branch, an accessory muscle, an atypical nerve communication, or a striking difference between the two sides of the same body, they begin to understand anatomy as a living biological field rather than a fixed catalogue.^20^ This experience also encourages humility. The body is not fully reducible to simplified diagrams. Clinical anatomy requires both knowledge of common patterns and readiness for individual difference.^13^

In this sense, donor‐based dissection supports a more mature form of anatomical reasoning. Students learn the standard arrangement, but they also learn that the standard arrangement is a reference point rather than an absolute guarantee.^18^ This is a crucial lesson for safe clinical practice.

## DISSECTION AS PREPARATION FOR CLINICAL REASONING

The value of anatomy in medical education is ultimately clinical. Anatomy becomes meaningful when it helps the physician understand symptoms, interpret images, perform procedures, anticipate complications, and explain disease processes.^2^, ^17^ Donor‐based dissection contributes to this goal when it is integrated with clinical reasoning rather than taught as isolated memorization.

Clinical reasoning often begins with localization. Where is the lesion? Which structure is compressed, inflamed, injured, obstructed, invaded, or denervated? Why does pain radiate to a particular region? Why does weakness follow a specific pattern? Why does fluid accumulate in one anatomical space rather than another? Why is a surgical approach safe in one plane and dangerous in another? These questions require an internal map of the body.

Dissection helps construct this map.^10^ In the upper limb, for example, students can relate the brachial plexus to the axilla, vessels, muscles, and potential compression sites. In the lower limb, they can understand compartmental organization, neurovascular bundles, fascial boundaries, and the anatomical basis of compartment syndrome, nerve injury, vascular access, and gait disturbance. In the abdomen and pelvis, they can appreciate peritoneal relationships, fascial planes, organ mobility, ureteric course, pelvic supports, and surgical corridors. In the head and neck, they can understand why small spaces have major clinical consequences.

Dissection also improves imaging interpretation. CT and MRI are not self‐explanatory. They require anatomical translation.^17^ A student who has seen structures in situ is better able to interpret sectional images because the image is connected to a three‐dimensional memory of the body. Similarly, ultrasound teaching becomes more meaningful when students understand the real depth, planes, and relationships beneath the probe.^21^

The educational goal, therefore, should not be to preserve dissection as a separate preclinical tradition. The goal should be to use dissection as a platform for clinical anatomical reasoning.^2^, ^10^ A well‐designed dissection session should ask students not only to identify structures but also to explain why those structures matter clinically.

## THE TACTILE AND MANUAL DIMENSION OF ANATOMICAL KNOWLEDGE

Much of modern anatomy education is visual. Students look at diagrams, screens, models, scans, and videos. Vision is essential, but anatomical knowledge is not purely visual. The hand also learns anatomy.^17^ Donor‐based dissection provides tactile and manual experiences that are difficult to reproduce digitally.

Through dissection, students learn the difference between fascia, fat, muscle, tendon, nerve, vessel, gland, membrane, and organ not only by appearance but also by texture, resistance, fragility, and behavior under manipulation.^2^, ^17^ They learn that a nerve can be delicate, a vessel can collapse, a fascial layer can be difficult to separate, a tendon can be firm and glistening, and fat can obscure important structures. They learn that blunt dissection and sharp dissection have different consequences. They learn that an anatomical plane is not simply a line in a diagram, but a physical interface that can be found and respected.

Beyond texture and resistance, dissection also provides an irreplaceable appreciation of physical properties that no two‐dimensional or three‐dimensional simulation can fully replicate, including the differences in weight and density between organs. The heaviness of the liver compared with the lightness of aerated lung tissue, for example, provides embodied anatomical knowledge that remains memorable and clinically meaningful. These differences in mass and density among the various organs of the body cannot be conveyed by any current digital platform.

This manual dimension has obvious relevance for future surgeons, but it is not limited to surgery.^17^ Emergency physicians, anesthesiologists, intensivists, radiologists, general practitioners, and many other clinicians perform procedures or interpret procedural anatomy. Venous access, arterial puncture, nerve block, lumbar puncture, thoracentesis, airway management, joint injection, wound exploration, and physical examination all depend on spatial and tactile understanding.

Digital tools can show where structures are. Dissection teaches how structures feel, how they are approached, how they can be damaged, and how they relate to the human body as material reality.^8^, ^14^ This distinction is educationally important. The hand learns anatomy differently from the eye.

## THE ETHICAL, EMOTIONAL, AND PROFESSIONAL DIMENSION

Donor‐based dissection is often one of the first moments in which medical students encounter the dead human body in an educational context. This experience has ethical, emotional, and professional significance.^22^, ^23^, ^24^, ^25^ The body of a donor is not merely an object of study. It is the body of a person who, through an act of altruism, has entered the educational life of future physicians. This fact introduces students to dignity, respect, responsibility, and the moral seriousness of medicine.

A well‐conducted dissection program teaches students how to balance scientific attention with human respect.^23^, ^24^ They must learn to examine, touch, dissect, and discuss the body without dehumanizing it. They must work as a team, follow laboratory rules, preserve structures, maintain cleanliness, and behave professionally. These behaviors are not secondary to anatomy; they are part of medical formation. The language used to describe those who donate their bodies matters as much as how their bodies are treated. Contemporary anatomy education increasingly favors person‐centered terminology—body donor, donated body, donor—precisely because it acknowledges the humanity, altruism, and educational contribution of the individual.^26^

The emotional dimension also deserves attention. Students may experience anxiety, curiosity, discomfort, detachment, gratitude, or reflection.^12^ These reactions should not be ignored. When guided appropriately, they can contribute to professional maturity. The dissection room becomes what Hildebrandt and colleagues have described as a safe space: an environment in which students begin to understand that medicine requires both knowledge and responsibility, and where they are supported to engage with mortality, uncertainty, and the ethics of care.^25^ They learn that the body is not simply a biological machine, but the physical presence of a human life.

The ethical formation supported by dissection extends to the broader professional identity of the physician. Interactions with body donors and their families, when facilitated appropriately, help students recognize the social contract that underpins medical education.^22^, ^25^ This sense of responsibility—to donors, to patients, and to the profession—is a core component of humanistic medical formation, and it cannot be fully reproduced by a digital model.^22^, ^27^ A virtual simulation can teach structure, but it cannot teach the same form of donor respect, mortality awareness, or professional comportment.^27^, ^28^

## LIMITATIONS OF DONOR‐BASED DISSECTION

A balanced account of dissection must acknowledge its limitations, which should not be minimized or ignored. Donor‐based anatomy teaching is expensive, time‐consuming, logistically demanding, and dependent on body donation availability.^29^ It requires specialized facilities, preservation systems, technical staff, health and safety measures, and trained educators. Preservation may alter tissue color, texture, and flexibility. Some regions may be small and complex (e.g., the pterygopalatine fossa) or difficult to dissect. Some donated bodies may be affected by disease, surgery, age‐related change, or preservation artifacts.

Dissection can also be pedagogically inefficient if poorly structured.^7^, ^10^, ^30^ Students may spend excessive time searching for structures without understanding their significance. They may become focused on technical exposure rather than anatomical reasoning. They may memorize labels without clinical integration. In large groups, some students may become passive observers rather than active participants. If tutors are not well‐trained, the session may become fragmented or reduced to a checklist.

There are also emotional and ethical responsibilities. Students must be prepared for the laboratory environment, supported if distressed, and educated in donor respect.^12^, ^29^ Institutions must maintain transparent and lawful body donation systems. Dissection should never be treated as a privilege detached from ethical obligation.

These limitations do not invalidate dissection, but they show that dissection is not automatically effective. Donor‐based dissection should be purposeful, structured, clinically integrated, and supported by complementary educational tools.^2^, ^7^ Tradition alone is not enough.

## DIGITAL ANATOMY, SIMULATION, AND ARTIFICIAL INTELLIGENCE: COMPETITORS OR COMPLEMENTS?

Digital anatomy and simulation technologies have transformed the educational landscape.^3^, ^14^ Their value should be recognized clearly. Interactive platforms can help students visualize complex regions, rotate structures, isolate systems, repeat difficult content, and prepare for laboratory sessions. Virtual dissection tables can provide standardized views and sectional correlations. Radiological databases can connect anatomy with clinical imaging. Ultrasound can bring living anatomy into the classroom.^21^ Three‐dimensional printing can support tactile learning of selected structures.^31^ Artificial intelligence can generate explanations, formative questions, personalized revision plans, and rapid feedback.^32^, ^33^

These tools are especially useful for reducing cognitive load before dissection.^8^, ^14^ A student who enters the laboratory with a preliminary understanding of the region is more likely to benefit from exploration of donated bodies. Digital resources can also help after the laboratory, allowing students to consolidate what they observed and correct misunderstandings. For small and/or complex regions such as the cranial base, pelvis, perineum, inner ear, pterygopalatine fossa, or autonomic pathways, digital visualization can provide orientation that is difficult to achieve through dissection alone.

However, digital tools also carry important limitations. They may present anatomy as overly clean, symmetrical, and simplified, potentially creating an illusion of mastery because structures are immediately visible, colored, and labeled.^8^ They may reduce the sense of depth, tissue resistance, and productive uncertainty. They may encourage passive observation if not linked to active learning tasks.^8^ Artificial intelligence tutoring tools, while increasingly capable, may generate confident but inaccurate anatomical explanations if not carefully supervised by expert educators.^32^, ^33^ The use of AI‐generated simulations of donor bodies raises additional ethical questions: Whether such simulations can or should replace the altruistic act of body donation, and whether the professional formation that arises from engaging with a real donated body can be achieved through virtual means.^27^, ^28^, ^34^ These are questions the anatomy education community is actively debating, and they deserve careful consideration rather than assumption in either direction.

The most productive approach is therefore not replacement but integration.^14^ Digital anatomy can prepare students for the dissection laboratory, and the dissection laboratory can make digital anatomy clinically meaningful. Simulation can support procedural rehearsal, while dissection can provide the anatomical reality behind the procedure. AI can assist learning, but anatomical educators must guide, verify, and contextualize its output.^32^, ^33^ In this integrated model, each method contributes what it does best.

## A PROPOSED HYBRID MODEL FOR CONTEMPORARY ANATOMY EDUCATION

The future of anatomy education should be based on a hybrid model that deliberately combines digital preparation, donor‐based experience, imaging correlation, clinical reasoning, reflection, and post‐laboratory consolidation.^4^, ^14^ Such a model avoids the false opposition between traditional and modern teaching and is consistent with contemporary educational theory. Kolb's experiential learning cycle — which posits that deep learning arises from concrete experience, reflective observation, abstract conceptualization, and active experimentation—maps well onto the sequence of phases described below.^4^ Each phase creates conditions for one or more components of this cycle, and together they constitute a constructively aligned anatomy curriculum in which all activities support the intended learning outcomes.^4^

The first phase is digital pre‐laboratory orientation. Before entering the dissection room, students should review the relevant region through atlases, videos, three‐dimensional platforms, imaging examples, and structured preparatory questions.^16^ Students may also benefit from a brief pre‐dissection reflection activity at this stage, which can help them emotionally prepare to encounter the donated body, clarify their expectations, and begin to engage with the ethical dimensions of donor‐based learning.^12^ The aim of this phase is not to replace the laboratory, but to reduce cognitive overload so that students arrive with a basic understanding of terminology, expected structures, regional boundaries, and learning objectives (Table 1).

**TABLE 1:** Educational dimensions of cadaveric dissection and digital anatomy tools.

Educational dimension	Cadaveric dissection	Digital anatomy, simulation, and AI	Best integrated use
Structural identification	Real structures in situ; variable and sometimes difficult to expose	Clear, labeled, repeatable visualization	Digital pre‐learning followed by cadaveric confirmation
Three‐dimensional reasoning	Real depth, continuity, and spatial complexity	Rotation, isolation, and layered visualization	Digital orientation plus cadaveric spatial exploration
Tactile learning	Tissue resistance, fragility, texture, and manual handling	Limited or absent tactile experience	Cadaveric/prosection‐based learning supported by models
Anatomical variation	Direct encounter with individual variation	Often standardized or idealized	Use cadaveric variation as a clinical teaching point
Clinical reasoning	Strong when integrated with cases and imaging	Strong for simulation and repeated scenarios	Combine dissection with clinical cases and imaging
Professional identity	Donor respect, teamwork, ethical seriousness	Limited ethical embodiment	Use reflection alongside cadaveric teaching
Revision and accessibility	Limited by laboratory access	Highly accessible and repeatable	Use digital tools for preparation and consolidation

The second phase is donor‐based or prosection‐based exploration. In this phase, students encounter real anatomical relationships. Depending on institutional resources, this may involve full dissection, guided dissection, prosection, plastinated specimens, or a combination of methods.^7^, ^10^, ^11^, ^30^ The essential point is that students must engage with real human anatomy, including depth, tissue planes, variation, and spatial continuity. Where full dissection is not feasible, rotating dissection systems—in which one group dissects and then presents their work to a second group before roles are reversed—offer a pedagogically valuable model that combines active exposure with peer teaching.^35^

The third phase is imaging correlation. Each regional anatomy session should be connected to relevant radiographs, CT images, MRI sections, ultrasound images, or endoscopic views.^21^ This helps students transfer knowledge from dissection into clinical interpretation. The donated body provides the anatomical reality; imaging shows how that reality appears in clinical practice.

The fourth phase is clinical case integration. Short, carefully designed cases can prompt students to use anatomy in reasoning. They should be asked where a lesion is likely to be, which structure is affected, what symptoms would follow, what anatomical variation might matter, and which procedure might be risky.^18^, ^20^ This transforms dissection from identification into applied reasoning.

The fifth phase is reflective and professional learning. Students should be given space to consider donor respect, teamwork, uncertainty, emotional response, and professional behavior.^12^, ^23^, ^25^ This may include memorial services for body donors, reading donor biographies or statements, and other humanistic approaches that acknowledge the altruistic contribution of those who donated their bodies for medical education.^12^, ^36^ Reflection need not be long or sentimental. Even brief structured reflection can help students recognize the ethical significance of donor‐based learning and begin to form the professional identity of a physician who understands both the science and the humanity of medicine.

The sixth phase is post‐laboratory digital consolidation. After the session, students can use digital tools, AI‐supported formative questions, annotated images, and structured revision tasks to reinforce the experience.^14^, ^32^ In this phase, technology extends the educational value of dissection beyond the laboratory.

Assessment should be integrated throughout this model rather than treated as a separate event. Importantly, assessment should include direct engagement with donor material through practical examinations, as well as technology‐based assessments using imaging, digital models, and clinical cases. The inclusion of donor‐based practical examination communicates to students that engagement with real human anatomy remains a core educational priority, one that technology should support but not supplant. Faculty should be explicit with students about the educational rationale for this balance: The latest technology is not a substitute for learning on the donated human body; it is a complement to it.

It should also be acknowledged that implementing this model is not without challenges. It requires institutional commitment of time and resources, adequate facilities, trained faculty, and sufficient body donation. Faculty development is a prerequisite: Tutors and demonstrators must be prepared not only as anatomical experts, but as facilitators of reflective, clinically integrated, and ethically aware learning experiences.^7^, ^10^ In settings where resources are constrained, a simplified version of the model—even if only three or four phases are implementable—may still yield educational benefit. The model should therefore be understood as a framework for aspiration and adaptation, not a prescriptive checklist.

## PRACTICAL RECOMMENDATIONS FOR ANATOMY EDUCATORS AND CURRICULUM DESIGNERS

Several practical recommendations follow from this argument for anatomy departments, course directors, laboratory demonstrators, and curriculum designers. First, donor‐based dissection should not be used merely because it has always been used. Each session should have explicit anatomical learning outcomes that identify not only structures to be found but also regional relationships, topographical orientation, variation, clinical relevance, and professional objectives.^2^, ^10^ Pre‐dissection reflection activities that help students emotionally and ethically prepare for the laboratory should be incorporated into session planning.^12^

Second, students should be prepared before entering the laboratory. Pre‐laboratory orientation, whether digital or through pre‐reading and lectures, can improve efficiency and reduce frustration.^16^ Students who understand the region beforehand are more likely to engage in meaningful exploration (Table 2).

**TABLE 2:** Practical recommendations for modern cadaveric anatomy teaching.

Educational challenge	Recommendation	Expected benefit
Reduced curricular time	Use focused, outcome‐based laboratory sessions	Greater efficiency and clearer purpose
Student cognitive overload	Provide digital pre‐laboratory orientation	Better preparedness and less confusion
Disconnection from clinical practice	Integrate imaging and short clinical cases	Improved clinical relevance
Passive student participation	Use guided tasks and active tutor facilitation	Greater engagement and reasoning
Limited cadaver availability	Combine prosections, plastination, digital tools, and selected dissection	Flexible preservation of real anatomy exposure
Overidealized anatomy learning	Highlight anatomical variation in cadavers	Better preparation for clinical reality
Ethical distancing	Include donor respect and brief reflection	Professional identity formation
AI‐related misinformation	Keep expert anatomical oversight	Safer and more accurate learning

Third, dissection should be integrated with imaging. Anatomy teaching that remains disconnected from CT, MRI, ultrasound, and radiographic interpretation risks appearing clinically remote.^21^ Imaging correlation helps students understand why anatomy matters.

Fourth, anatomical variation should be explicitly taught. When variation is encountered, it should be discussed as a clinically important reality rather than treated as a distraction from the standard pattern.^18^, ^19^, ^20^ A curriculum framework that treats variation as the norm rather than the exception provides students with a more accurate and clinically relevant understanding of human anatomy.^19^

Fifth, clinical cases should be embedded into laboratory teaching. These cases need not be long. Even brief prompts can guide students from structure identification to clinical reasoning.^17^

Sixth, tutors and demonstrators should be trained in active facilitation. The role of the tutor is not simply to point out structures or answer questions. It is to guide observation, stimulate reasoning, correct misconceptions, and connect anatomy to future clinical practice.^7^, ^10^

Seventh, prosections and digital tools should be used strategically where full dissection is not feasible. The goal is not to insist on one format for every institution, but to preserve meaningful contact with real human anatomy.^11^, ^14^, ^30^

Eighth, donor respect and professional conduct should be made explicit. Students should understand the ethical basis of donor‐based learning and the responsibilities that accompany it.^24^, ^25^, ^29^ The language used in the classroom and the laboratory should reflect person‐centered values: referring to the individuals who donated their bodies by terms that acknowledge their humanity and altruism.^26^

Finally, artificial intelligence should be used carefully. AI can support preparation, revision, and formative assessment, but it should not be allowed to replace expert anatomical supervision.^32^, ^33^ Inaccurate anatomical explanations can be educationally harmful, especially when presented with confidence. The broader ethical questions raised by AI simulation of human bodies in anatomical education deserve ongoing scholarly attention.^27^, ^28^

## CONCLUSION

Donor‐based dissection remains indispensable in medical education when its role is properly understood. It is not indispensable because it is the only way to learn anatomical facts. Many facts can now be accessed through atlases, digital models, imaging platforms, videos, simulations, and artificial intelligence tools.^8^, ^14^ It is indispensable because it provides a form of learning that is spatial, tactile, variable, ethical, emotional, professional, and clinically meaningful.

The donated body teaches anatomy as the body truly is: layered, continuous, individual, and sometimes uncertain.^2^ It challenges the simplifications of diagrams and digital models. It teaches students to discover structures, respect tissue planes, recognize variation, build three‐dimensional maps, and connect anatomical knowledge to clinical reasoning. It also introduces them to donor respect, mortality, teamwork, and the seriousness of medical responsibility.^22^, ^23^, ^24^, ^25^

The future of anatomy education should not be framed as a struggle between the donated body and technology. Digital anatomy, simulation, imaging, ultrasound, 3D models, and artificial intelligence all have roles.^14^ Their greatest value may be achieved when they are integrated with donor‐based learning rather than used to replace it. The goal should be a coherent educational ecosystem in which students prepare and pre‐learn, then learn with the body donors, translate that knowledge through imaging and clinical cases, reflect on professional meaning, and consolidate their understanding through modern learning tools.

In this sense, donor‐based dissection is not a relic of the past. It is a continuing foundation for clinically grounded anatomical education. Its role should be refined, integrated, and pedagogically strengthened—not abandoned.^2^, ^4^

## AUTHOR CONTRIBUTIONS

**Uğur Baran Kasırga:** Conceptualization; visualization; writing – original draft; writing – review and editing.

## FUNDING INFORMATION

This research received no specific grant from any funding agency in the public, commercial, or not‐for‐profit sectors.

## CONFLICT OF INTEREST STATEMENT

The author declares no conflict of interest.

## ETHICS STATEMENT

This article is a review and educational perspective. No human participants, patient data, or animal subjects were involved in its preparation. No ethics approval was required.

## ARTIFICIAL INTELLIGENCE DISCLOSURE STATEMENT

Artificial intelligence writing assistance (Claude, Anthropic) was used in drafting and editing portions of this manuscript. The author reviewed, verified, and takes full responsibility for all content. No AI tool was used to generate figures or illustrations.

## Data Availability

Data sharing is not applicable to this article as no new data were created or analyzed in this study.
